# Development of the 7‐item ASK‐AD Assess Symptoms and Knowledge of Alzheimer’s Disease tool for multigenerational community dementia awareness: Findings from Old SCHOOL Hip Hop

**DOI:** 10.1002/alz.089722

**Published:** 2025-01-09

**Authors:** James M. Noble, Sandra G Minchala, Monique G Hedmann, Jeanne Teresi, Katja Ocepek‐Welikson, Stephanie A Silver, Joseph P Eimicke, Mildred Ramirez, Adrian Harris, Arthur Lloyd, Kunsorya Chhea, Catalina Alvarez Arango, Yejin Lee, Vanessa Sawyer, Olajide Williams

**Affiliations:** ^1^ Columbia University Irving Medical Center, New York, NY USA; ^2^ Massachusetts General Hospital, Boston, MA USA; ^3^ Harlem Hospital Center, New York, NY USA

## Abstract

**Background:**

Alzheimer’s disease (AD) health literacy is low in high‐risk populations and is likely a determinant of timely care seeking behavior. Our group aimed to develop a novel brief questionnaire for use in community outreach and related studies of AD awareness.

**Methods:**

We developed an initial 15‐item AD knowledge questionnaire “ASK‐AD (Assess Symptoms and Knowledge of AD)” following pilot study and cognitive interviews with subject matter experts along with elementary school children. The study took place in 28 elementary schools in the New York City area between 2018‐2023. Psychometric analyses explored data acquired immediately following enrollment but before randomization into either arm of the Old SCHOOL Hip Hop randomized controlled trial.

**Results:**

Overall, 1461 diverse elementary school children including 692 (47%) fourth graders and 752 (52%) fifth graders (typically aged 9‐11 years) enrolled along with 783 of their parents. Among parents, 679 (87%) were female with 441 (56%) self‐reporting Black or African‐American race and 215 (28%) Hispanic ethnicity; 97 (12%) reported White race. Most parents were aged 36‐55 years (521 (67%), 18‐35y: 223 (29%)) with 185 (24%) having completed high school; 226 (29%) completed some college, and 182 (24%) had a bachelor’s degree. Performance metrics of 15‐, 11‐, 8‐, and 7‐ question versions were explored. The 7‐item version of the ASK‐AD evidenced optimal performance in students and parents, testing key AD concepts, including common symptoms and signs (six items) and brain localization (one item). ASK‐AD‐7 corrected item‐total correlations ranged from 0.30‐0.50 for student data and 0.24‐0.53 for parents. Psychometric property estimates were good‐to‐excellent for parent and student versions. The unstandardized Cronbach’s alpha coefficient estimate was 0.668 (standardized = 0.667) for students and 0.699 (0.701) for parents. For parents, the McDonald’s Omega estimate Total (ωt) was 0.857, ordinal alpha was 0.854, and the explained common variance (ECV) was 59.567; for students ωt = 0.801, ordinal alpha = 0.798 and ECV = 49.338. Items of AD prevention, treatment, and action plan were not retained in any reduced version.

**Conclusions:**

The novel 7‐item ASK‐AD multigenerational community dementia awareness tool may be used in diverse, at‐risk groups. ASK‐AD could fill important gaps in ongoing AD community outreach programs.

